# Future trends of life expectancy by education in the Netherlands

**DOI:** 10.1186/s12889-022-13275-w

**Published:** 2022-09-02

**Authors:** Wilma J. Nusselder, Anja M. B. De Waegenaere, Bertrand Melenberg, Pintao Lyu, Jose R. Rubio Valverde

**Affiliations:** 1 Department of Public Health - Erasmus Medical Center, Rotterdam, the Netherlands; 2 Tilburg School of Economics and Management, Department of Econometrics and Operations Research, Tilburg, the Netherlands

**Keywords:** Mortality projection, Socioeconomic position, Life expectancy

## Abstract

**Background:**

National projections of life expectancy are made periodically by statistical offices or actuarial societies in Europe and are widely used, amongst others for reforms of pension systems. However, these projections may not provide a good estimate of the future trends in life expectancy of different social-economic groups. The objective of this study is to provide insight in future trends in life expectancies for low, mid and high educated men and women living in the Netherlands.

**Methods:**

We used a three-layer Li and Lee model with data from neighboring countries to complement Dutch time series.

**Results:**

Our results point at further increases of life expectancy between age 35 and 85 and of remaining life expectancy at age 35 and age 65, for all education groups in the Netherlands. The projected increase in life expectancy is slightly larger among the high educated than among the low educated. Life expectancy of low educated women, particularly between age 35 and 85, shows the smallest projected increase. Our results also suggest that inequalities in life expectancies between high and low educated will be similar or slightly increasing between 2018 and 2048. We see no indication of a decline in inequality between the life expectancy of the low and high educated.

**Conclusions:**

The educational inequalities in life expectancy are expected to persist or slightly increase for both men and women. The persistence and possible increase of inequalities in life expectancy between the educational groups may cause equity concerns of increases in pension age that are equal among all socio-economic groups.

**Supplementary Information:**

The online version contains supplementary material available at 10.1186/s12889-022-13275-w.

## Introduction

Increased longevity poses great challenges to the welfare state, including the sustainability of pension systems. In response to these challenges, several countries introduced changes in the pension system and increased the retirement age [1, 2]. According to the OECD, around two-thirds of reforms automatically linked future pensions to (projected) changes in life expectancy. Some countries adjust benefit levels to life expectancy (Germany, Finland, and Portugal), other countries link the number of years of contributions needed for a full pension to life expectancy (France), whereas again in other countries the pension age is linked to the increase in life expectancy (the Netherlands, Denmark, Estonia) [2, 3]. For these and other purposes, national projections of future mortality are made periodically by statistical offices or actuarial societies in Europe. Most projections are based on extrapolative approaches, with the Lee-Carter method mostly used [4–6]. The Lee-Carter model summarizes mortality by age and period for a single population as an overall time trend, an age profile, and the age-specific deviations of mortality change over the entire fitting period [7]. Some recent national projections include mortality data from neighboring countries to increase the robustness of the projections using the Lee and Li approach [5, 6, 8].

Linkage of future pensions to the life expectancy of the national population might have different consequences for different socioeconomic groups, because of differences in mortality within the national population. People with a lower level of education on average have higher mortality than people with a higher level of education [9]. Inequalities in mortality translate into substantial inequalities in life expectancy. For example, in the Netherlands the gap in period life expectancy at birth between high and low educated is 6.3 years for men and 3.3 years for women [10].

National projections of life expectancy may not provide a good expectation of the future trends in life expectancy of different social-economic groups. First, there is no guarantee that trends in mortality of different socioeconomic groups are parallel or converging to a common overall trend. Over the past two to four decades relative inequalities in mortality have increased in almost all European countries, whereas absolute inequalities in mortality trends have followed a more variable course [9, 11–13]. Moreover, trends in equalities have been shown to differ depending on the mortality measures and inequality measures that are used [9].

Second, even in the situation of equal trends in mortality rates of different socioeconomic groups, this may not translate into equal trends in life expectancy of these groups. Paradoxically, an increasing gap in life expectancy between socioeconomic groups may even arise in the situation of an identical drop in mortality rates for each group. Such an identical absolute drop in mortality rates increases life expectancy of the higher socioeconomic group more because of the higher ‘ex post survivability’, i.e., as compared to lower socioeconomic groups, higher socioeconomic groups have lower mortality rates at ages above the ages at which the drop occurred. Moreover, people from lower socioeconomic groups are less likely than those from higher socioeconomic groups to survive long enough to benefit from the reduction in mortality that occurs at older ages (‘ex ante survivability’) [14]. This implies that an equal reduction in mortality of low and high educated may translate into a larger life expectancy increase of the higher socioeconomic group.

Third, when socioeconomic status is measured by education and different educational groups have identical trends in life expectancy, this common trend will not be equal to that of the national population. Because of educational expansion, i.e., a growing part of the population having a higher education and a reducing part having a lower education, the increase in life expectancy at the national level is partly due to changes in the educational composition. As a consequence, even in the unlikely situation of zero change in life expectancy of each educational subgroup, life expectancy of the national population will increase due to educational expansion. Similarly, identical non-zero trends in life expectancy of each educational subgroup yield larger increases in life expectancy of the national population than for each subgroup. Luy et al. [15] estimated that the change in educational composition between around 1990 and 2010 accounted for approximately one year of the increase in life expectancy at age 30 in Italy and Denmark, and about 0.5 year in the United States. This corresponds to 19.1% of the total increase in that period for Italy, 19.9% for the US, and 24% for Denmark.

Because future trends in life expectancy for different socioeconomic subgroups cannot be assumed to be same as for the national population, there is an urgent need for mortality projections for different socioeconomic groups. To date, projections of mortality and of resulting life expectancy for different socioeconomic groups are scarce. Some exceptions are a recent projection of life expectancy at birth for different income groups for South Korea [16], projections of remaining life expectancy for socioeconomic groups in Denmark based on an individual affluence index [17], and a projection of life expectancy at age 65 for different education groups for the Netherlands, published five years ago [18]. In addition, there are a few projections by deprivation or wealth index of small areas [19, 20], a study that models mortality for socioeconomic groups but does not produce forecasts [21], and a study that models and projects mortality for different socioeconomic groups but does not present forecasts of future life expectancies of these groups [22].

Two reasons may explain the scarcity of mortality projections for different socioeconomic groups. First, the availability of time-series data of mortality by socioeconomic group, age and gender is limited, and if available time series are generally shorter than routinely used for mortality projections. Second, independent extrapolations of mortality for separate socioeconomic groups can lead to inconsistent results across the subgroups because it ignores common factors that may affect all subgroups. Mortality projections by subgroup require more complex approaches, such as the Lee and Li approach [23], that account for those common factors and that produce coherent projections for the different subgroups. The original Li and Lee approach uses mortality data of several countries to create a broad empirical basis for the identification of the most likely long-term common trend combined with country-specific deviations from that common trend [8, 24, 25]. It is currently used to project national mortality rates for Belgium and the Netherlands, using data from multiple countries [6]. The combination of the multi-country approach and different socioeconomic groups that we develop in our study is a natural next step that allows us to maximally use available data to make stable projections for educational groups.

The objective of this study is to derive insight in future trends in life expectancies for low, mid and high educated men and women living in the Netherlands. To improve the robustness of the extrapolations and to include information on longer time trends of mortality than the relatively short time series by education in the Netherlands, we use a three-layered Lee and Li approach. As upper layer we use national mortality data by age and gender in the Netherlands and five other North-Western European countries for the period 1970–2018, as second layer we use education-specific mortality by age and gender from these countries per 5-year periods for the period 1990–2015, and as third layer we use mortality by education, age and gender per year for the Netherlands for the period 2006–2018.

## Methods

### Data

Mortality data by education for the Netherlands were obtained through individual data linkage of register data of all persons living in the Netherlands, within the secure environment of Statistical Netherlands. A file with individual data of all persons based on the population registry (‘Basis Registratie Personen’, BRP), was linked to a data file with anonymized codes of addresses, and begin and end of each period a person lived at a particular address (to exclude person-years not lived in the Netherlands), and a data file with deaths of persons living in the Netherlands. Data on the educational attainment was based on the Educational Attainment File derived by Statistic Netherlands by combining information on education levels from several registries, including educational registries and unemployment registries, and from Labor Force Surveys. The educational attainment file started with data from 1999 onwards, but coverage was increasing over time to 11 million persons in 2018 (out of 17.1 million inhabitants). As there is no information on educational attainment for every citizen in the population, weights in combination with a calibration procedure developed by Statistical Netherlands [26] were used to ensure that the mortality rates by education based on linkage with educational registers are representative for the Dutch population. We derived mortality rates by education for age groups 35–39,…,80–84, per calendar year for the period 2006–2018.

In addition, we used mortality data by age, gender and education for variable periods between 1990 and 2015 for five other North-Western European countries, i.e., Finland, Norway, Denmark, Belgium, and Switzerland. These data were collected and harmonized as part of European projects at Erasmus MC and described in prior publications [27, 28]. More details on the mortality data are given in Appendix 1 The five countries were selected because these countries provided data based on individual mortality follow up, had sufficiently long time series, provided data related to national populations and included three similar educational groups (low, mid and high educated). We excluded countries meeting these criteria with large educational inequalities (in Eastern and Central Europe) and small educational inequalities (in Southern Europe). Using mortality derived from individual mortality follow-up, avoids bias in unlinked data [29] also known as dual-data-source bias [30]. Data were available in 5-year age groups and per 5-year calendar-period and three educational groups (low, mid and high). The five countries did not include exactly the same 5-year periods. We calculated for each country the midpoint of each 5-year period and we selected as common midpoints the years 1993, 1998, 2003, 2008 and 2013. This selection minimizes changes in the composition of the group of countries for which data is used over time. In case a calculated midpoint did not match exactly with one of the common midpoints, we shifted the calculated midpoint to the closest common midpoint (e.g. for Belgium the calculated midpoint was 1994 which was shifted to 1993). The shifts were maximum one year. For a summary of the data and the allocation to common midpoints, see Table 1 in the model description in Appendix 2.

Finally, we used on mortality by age and gender for the period 1970–2016 from the Human Mortality Database (HMD) [31]. We included data on deaths and person-years (i.e., exposures) by 5-year age group for the age range 35–39 and over.

### Socioeconomic Position indicator

Educational attainment is used as measure of socioeconomic position. Educational attainment is usually completed in early adulthood, which largely avoids the problem of reverse causation in studying adult mortality (i.e. low education as the result of poor health or health losses) [32]. This is in contrast with for example income, which may also be the result of poor health or health losses, and may change substantially over the life course. A second reason to use mortality data by education is that such data, classified in a comparable format based on the international ISCED classification [33], could be obtained for several European countries based on individual mortality follow-up. Level of education was categorized into three levels: low (ISCED 0–2), medium (ISCED 3–4), and high (ISCED 5 +).

### Statistical analyses

#### Modelling and predicting mortality rates

To model Dutch education-specific mortality, we use a three-layer Li and Lee model [23]. The upper layer models a common trend of log mortality for all countries included in the HMD database^1^ and all educational levels (referred to as `HMD’ mortality). The second layer models the deviation of education-specific log mortality of selected European countries (referred to as ‘INT EDU’ mortality) from the HMD mortality. The third layer models the deviation of Dutch education-specific log mortality (referred to as ‘NL EDU’ mortality) from the INT-EDU mortality. Hence, Dutch education-specific log mortality is the sum of the three layers. Each of the three layers is modeled using the Lee and Carter approach [7]. The Lee-Carter model summarizes mortality by age and period for a single population as a function of an age-effect, a time trend, and age-specific sensitivities to the time trend [1]. In contrast with the original Li and Lee model, we do not impose that education-specific mortality convergences to the common trend. Moreover, we use the modified estimation method of Liu et al. [34] to estimate the model parameters. Further information about the estimation of the three-layered Li and Lee model and the selection of time series processes is presented in Appendix 2.

We estimated the model separately for men and women for the age range 35–85 years to derive age, sex and education specific mortality rates. Because of lower data quality of mortality by education at older ages, we extrapolated mortality rates above age 85 using a modification of the Kannisto method [35]. For a detailed description, see Appendix 2.

### Construction of life tables

We constructed period life tables by gender for low, mid and high educated men and women based on the estimated and projected mortality rates. We used standard life table methods for abridge life tables (i.e. 5-year age groups), assuming constant death rates within the age interval [36]. We present life expectancy by education between age 35–85 years (i.e., partial life expectancy), and remaining life expectancy by education at age 35 and at age 65. Remaining life expectancy includes extrapolated mortality rates for age 85–89 up to and including age 110 + . Partial life expectancy, also known as temporal life expectancy, refers to the expected number of years lived between specific ages, in this case the ages of 35 and 85. The maximal number of years lived between ages 35 and 85 is 50 years. The partial life expectancy is strictly below 50 years because several persons die between their 35th and 85th birthday. Results on partial life expectancy are often presented because partial life expectancy is a measure that can exclude age ranges where data may be less reliable or absent [37], or because one wants to focus on a specific age range relevant for the topic under study, such as ages around the retirement age [38]. We present partial life expectancy for the age range 35 to 85 because this matches the range for which we have mortality data by education. In Appendix 3 we also present partial life expectancy for the age range 35 to 80 to allow for comparison with other studies on educational inequalities that use this age range [9, 39, 40]. We present outcomes for three time points, 15 years separated: 2018 (last year of observation), 2033 and 2048. In addition, we present the change in **(**partial and remaining**)** life expectancy as compared to 2018 and the change per year (change / number of years). Life expectancy estimates for other years are presented in Appendix 4.

### Educational differences

We use the difference in life expectancies between the high and low educated, expressed in years, as the inequality measure. Because absolute differences in life years lost and in life expectancy are the same, our inequality measure can also be interpreted as difference in years lost.

Data preparations were done in STATA, version 16, model estimation and projection of mortality rates in Matlab, version R2019b, time series estimation in R, version X62 462, and calculations of life expectancies and their inequalities in Excel 2016.

## Results

### Mortality trends

**Fig. 1:**
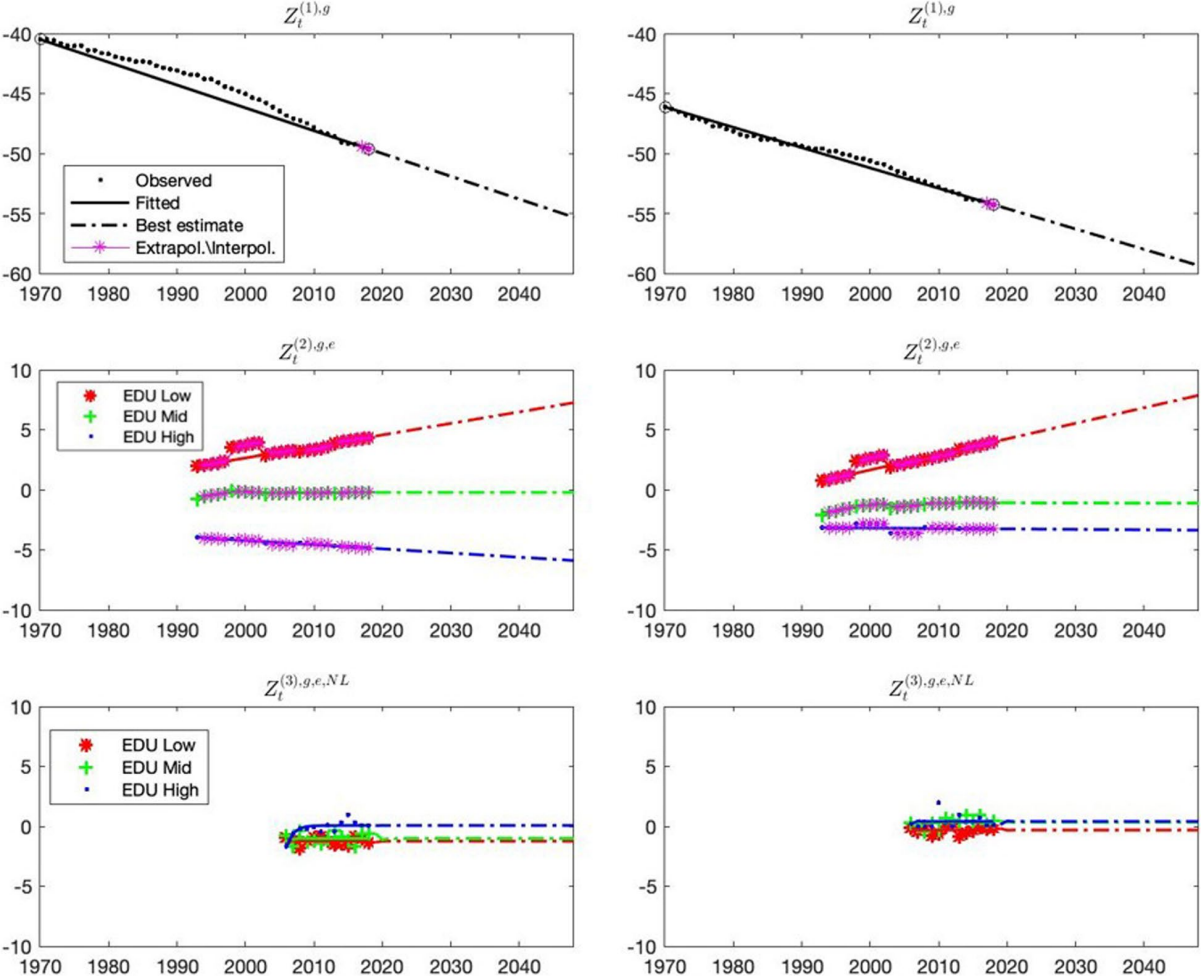
The top left (right) panel displays the development over time of the aggregate log mortality of men (women) in the HMD population with all educational levels combined. The middle panels (left for men and right for women) display the development over time of the difference between education-specific aggregate log mortality in the international population (INT-EDU) and aggregate log mortality in the HMD population with all educational levels combined. Red corresponds to the low education group, green to the mid education group, and blue to the high education group. A positive value implies that the educational group has higher mortality rates than the HMD population with all educational levels combined; a negative value implies that it has lower mortality rates. The bottom left (right) panel displays the development over time of the difference between education-specific aggregate log mortality rates in the Dutch population (NL-EDU) and education-specific aggregate log mortality rates in the international population (INT-EDU), for the three educational levels. A positive (negative) value for an educational group implies that the mortality rates of that educational group in the Dutch population (NL-EDU) are higher (lower) than the mortality rates of the same educational group in the international population (INT-EDU). In all six panels, the dots represent observed values, the solid lines represent the fitted values in our Li and Lee model, the pink stars represent extrapolated or interpolated values, and the dashed-dotted lines represent best-estimate model forecasts. In terms of the Eqs. (11) and (12) in Appendix 2, the top panels correspond to the variables Z_t_^1,g^ in the first layer, the middle panels correspond to the variables Z_t_^2,g,e^ in the second layer, and the bottom panels correspond to the variables Z_t_^3,g,e,NL^ in the third layer

Figure 1 displays the common trend in the aggregate (over ages) log mortality for the international population with all educational levels combined (i.e., ‘HMD’ mortality, top panels), the deviation of international education-specific aggregate log mortality (i.e., INT-EDU mortality) from this common trend (middle panels), and the deviation of Dutch education-specific aggregate log mortality (i.e., NL-EDU mortality) from international education-specific aggregate log mortality (bottom panels). One obtains the Dutch education-specific aggregate log mortality by adding these three layers.

The top panels in Fig. 1 show that for both men and women, the aggregate log mortality trend in the HMD population with all educational levels combined is decreasing over time, indicating a decline in mortality. The middle panels in Fig. 1 show the trend in the difference between aggregate log mortality rates in the INT-EDU and the HMD population, for the three educational levels and for both genders. We observe the following:

- Low education group (middle panels, red lines/dots/dashes): for both genders, the difference between INT-EDU aggregate log mortality rates and HMD aggregate log mortality rates is positive and increases over time. This indicates that mortality rates of low educated men (women) in the international population are higher than mortality rates of the HMD population without distinction by education, and that these differences increase over time.
- Mid education group (middle panels green lines/dots/dashes): for both genders, the difference between INT-EDU aggregate log mortality and HMD aggregate log mortality is close to zero and relatively flat over time, indicating that the trend of mortality of mid educated men (women) in the international population is similar to the trend in the HMD population without distinction by education.
- High education group (middle panels, blue lines/dots/dashes): for high educated men, the difference between INT-EDU aggregate log mortality rates and HMD aggregate log mortality rates is negative and decreasing over time, indicating that mortality rates of high educated men of the international population are below those of the HMD level for men, and that the difference is becoming bigger over time. For high educated women, the results are similar but the difference between international education-specific mortality rates and mortality rates in the HMD population increases at a much smaller rate than for men.

Finally, the bottom panels in Fig. 1 display the deviation between Dutch education-specific aggregate log mortality rates (NL-EDU) and international education-specific aggregate log mortality rates (INT-EDU) for men and women and for the three education groups. The panels show that in all six cases, the deviation of the Dutch education-specific mortality rates from the international education-specific mortality rates is very small and fluctuates around zero with no clear trend over time.

### Life expectancies by education

Table 1 presents the current and future period life expectancy between age 35 and 85 for low, mid and high educated, based on the estimated and forecasted mortality rates between age 35 and 85, for the last observation year (2018) and two future years (2033 and 2048) yielding three time points 15 years apart. In addition, the change as compared to 2018 and the change per year (change / number of years) are presented. Appendix 3 gives the life expectancies for the complete set of years between 2006 and 2048. For the years 2006–2018, also a comparison is made between life expectancy between age 35 and 85 based on observed death probabilities and based on modelled death probabilities (Appendix 5). This shows a large similarity between both life expectancies, i.e., our model fits the data quite well.

Life expectancy between age 35 and 85 increases over time in all educational groups and for both genders. For low educated men, it is projected to increase from 41.7 years in 2018 to 43.4 years in 2048, an increase of 1.7 years in 30 years (annual change of 0.06 years). For high educated men, it is projected to increase from 45.3 years in 2018 to 47.2 years in 2048, an increase of 1.9 years (annual change of 0.06 years). For women the projected increases are smaller, and are particularly small for lower educated women. In that group, partial life expectancy is projected to increase from 44.1 years to 44.5 years, an increase of 0.4 years in 30 years (annual increase of 0.01). For high educated women, it is projected to increase from 46.6 years in 2018 to 47.9 years in 2048, an increase of 1.3 years in 30 years (annual increase of 0.04).

Table 1 presents also the remaining life expectancy at age 35 and age 65 for the years 2018, 2033 and 2048. For low educated men, remaining life expectancy at age 35 is projected to increase with 2.8 years in 30 years (0.09 years annually) and for high educated men with 4.1 years (0.14 years annually). For low educated women, remaining life expectancy at age 35 is projected to increase with 1.8 years (0.06 years annually) and for high educated women with 3.5 years (0.12 years annually).

**Table 1:** Life expectancy (LE) between age 35–85 and remaining life expectancy at age 35 and at age 65 by gender and education (in years)

	Low	Change since 2018		Mid	Change since 2018		High	Change since 2018	
		Total	Per year		Total	Per year		Total	Per year
**Men**									
LE35-85									
2018	41.7			43.7			45.3		
2033	42.6	0.9	0.06	45.2	1.5	0.10	46.4	1.1	0.07
2048	43.4	1.7	0.06	46.3	2.6	0.09	47.2	1.9	0.06
LE 35									
2018	43.6			46.0			48.7		
2033	44.8	1.2	0.08	48.4	2.4	0.16	50.8	2.1	0.14
2048	46.3	2.8	0.09	50.4	4.4	0.15	52.8	4.1	0.14
LE 65									
2018	17.3			18.5			20.5		
2033	18.3	1.0	0.07	20.4	1.8	0.12	22.2	1.7	0.12
2048	19.7	2.4	0.08	22.0	3.4	0.11	23.9	3.5	0.12
**Women**									
LE35-85									
2018	44.1			45.8			46.6		
2033	44.3	0.4	0.02	46.7	0.9	0.06	47.3	0.7	0.04
2048	44.5	0.4	0.01	47.4	1.6	0.05	47.9	1.3	0.04
LE 35									
2018	47.5			50.3			51.5		
2033	48.3	0.8	0.06	52.4	2.1	0.14	53.1	1.6	0.011
2048	49.3	1.8	0.06	54.4	4.2	0.14	55.0	3.5	0.12
LE 65									
2018	20.7			22.3			23.0		
2033	21.7	1.0	0.07	24.1	1.8	0.12	24.4	1.4	0.09
2048	23.0	2.3	0.08	25.9	3.6	0.12	26.1	3.1	0.10

Remaining life expectancy at age 65 is projected to increase with 2.4 years in 30 years (0.08 years annually) for low educated men and with 3.5 years (0.12 years annually) for high educated men. For low educated women, life expectancy at age 65 is projected to increase with 2.3 years (0.08 years annually) and for high educated women with 3.1 years (0.10 years annually).

### Educational differences

Table 2 shows the educational differences in life expectancy between age 35 and 85 and in remaining life expectancy at age 35 and at age 65 for 2018, 2033 and 2048, showing similar or slightly increasing inequalities over time. The inequality in life expectancy between age 35 and age 65 was 3.6 in 2018 and for the year 2048 this is projected to be similar (3.8 years). For women, the inequality in life expectancy between age 35 and age 85 was 2.6 years in 2018 and is projected to be around 3.4 in 2048, an increase of 0.8 years (annual increase of 0.03). The difference in life expectancy at age 35 between low and high educated men was 5.1 years in 2018 and is projected to be around 6.4 years in 2048, an increase of 1.4 years (annual increase of 0.05 years). For women this is 4.0 years in 2018 and projected to be around 5.7 years in 2048, an increase of 1.7 years (annual increase 0.06 years). At age 65, for men, the inequality increases slightly from 3.2 years in 2018 to 4.3 years in 2048, an increase of 1.1 (annual increase of 0.04 years) and for women from 2.4 to 3.2, an increase of 0.8 (annual increase 0.03).

**Table 2:** Educational differences (high-low) in life expectancy (LE) between age 35–85 and in remaining life expectancy at age 35 and at age 65, by gender and education (in years)

	Difference	Change since 2018		Difference	Change since 2018	
		total	per year		total	per year
Men						
LE35-85						
2018	3.6			2.0		
2033	3.9	0.2	0.01	2.6	0.6	0.04
2048	3.8	0.2	0.01	2.8	0.8	0.03
LE 35						
2018	5.1			2.4		
2033	6.0	0.9	0.06	3.6	1.2	0.08
2048	6.4	1.4	0.05	4.1	1.7	0.06
LE 65						
2018	3.2			1.2		
2033	3.9	0.7	0.05	2.0	0.8	0.05
2048	4.3	1.1	0.04	2.3	1.1	0.04
Women						
LE35-85						
2018	2.6			1.8		
2033	3.0	0.4	0.03	2.4	0.6	0.04
2048	3.4	0.8	0.03	2.9	1.2	0.04
LE 35						
2018	4.0			2.8		
2033	4.8	0.8	0.05	4.1	1.3	0.09
2048	5.7	1.7	0.06	5.1	2.3	0.08
LE 65						
2018	2.4			1.7		
2033	2.8	0.4	0.03	2.5	0.8	0.05
2048	3.2	0.8	0.03	2.9	1.3	0.04

## Discussion

### Main findings

Based on our forecasts of future mortality rates derived with a Li and Lee model, we predict that increases of life expectancy between age 35 and 85 and of remaining life expectancy at age 35 and age 65 will continue in the future for all education groups in the Netherlands. The projected increase in life expectancies is larger among the high educated than among the low educated. Life expectancy of low educated women, particularly between age 35 and 85, shows the smallest projected increase. Our projections suggest inequalities in life expectancies at age 35 between high and low educated to be close to constant or slightly increasing over time. Our projections also suggest educational inequalities in life expectancy to remain larger among men than among women, but women to be catching up in terms of inequalities, particularly for life expectancy between age 35 and 85 and remaining life expectancy age 35.

The persistence of inequalities in life expectancy between educational groups should not be seen in isolation from the compositional change characterized by a reduction of the proportion of the population with lower education and an increase of the proportion of the population with a higher education. This process of educational expansion is expected to continue in the Netherlands in the period covered by our projection. In 2019, the percentage of high educated in the age group 25 to 65 years was approximately 35 percent and is projected to increase to 44 percent in 2050. For the 80 + age group, less than 20 percent was higher educated in 2919, and this percentage is expected to increase to 30 percent in 2050 [41]. Clearly, education expansion has a direct effect on mortality projections for national populations, with population rates becoming more strongly determined by those of the high educated. There is some evidence that changes in the educational distribution are also associated with changes in education-specific mortality [42–44]. One of the reasons is that in lower educated groups, there is an increased concentration of disadvantage in terms of personal characteristics which are also related to mortality. In our Li and Lee framework, all time dependent factors that affect mortality rates of the population and/or education groups (including educational expansion) are captured in the time variables Z_t_^1,g^, Z_t_^2,g,e^, and Z_t_^3,g,e,NL^ in the three layers. Past trends are estimated and projected into the future using best-estimate projections.

The persistence of inequalities in life expectancy between low and high educated in our projections is plausible if we consider expected trends in determinants of educational inequalities in life expectancy. A recent study of Mackenbach et al. [39] showed that smoking, low income and high body weight are risk factors that contribute most to inequalities in life expectancy between ag 35 and 80. The Dutch National Institute of Public Health [45] expects that diverging trends of smoking and obesity prevalence between educational groups, currently witnessed in the Netherlands, will continue in the future. The least favorable mortality trends for low educated women may be partly related to smoking. This hypothesis is in line with the model of the ‘smoking epidemic’ that describes differences in the progression of the smoking epidemic between genders and socioeconomic groups [46]. It is also in line with results from a comparative study of several European countries that shows larger declines in mortality from smoking-related causes of death for high than for low educated women, whereas the reverse is true for men [28]. Finally, smoking-attributable mortality has been shown to decrease in consecutive birth cohorts among low- and high educated men and high-educated women, but not among low educated women in whom it increased [47].

### Strengths and limitations

A strength of our study is that we used a coherent forecasting method, developed by Li and Lee [23] to model different educational groups, using linked mortality data. Dual data sources to compute education-specific mortality have been shown to overestimate mortality among the low educated and overestimate educational differences in mortality and may cause or exaggerate convergence in mortality between high and low educated [30]. By using linked mortality data, we avoid the numerator-denominator bias present in unlinked data sources.

The studies that are closest to ours are Khang et al. [16] who use independent Lee Carter models to project life expectancy at birth for different income groups for South Korea, Kjærgaard et al. [17] who use a CoDA approach (a Lee and Li type approach based on life table deaths instead of death rates) to project remaining life expectancy for socioeconomic groups based on an individual affluence index in Denmark, and Van Baal et al. [18] who use a Lee and Li approach to project life expectancy at age 65 for different education groups for the Netherlands. By using independent Lee Carter models, the approach in Khang et al. ignores (likely) dependence between different socioeconomic groups caused by common factors (e.g., healthcare improvements) that affect all groups. Kjærgaard et al. account for this dependence but rule out widening gaps between socioeconomic groups by imposing that the trends in the mortality improvements of different socio-economic groups converge to a common trend. Like Van Baal et al., we mitigate these concerns by using a Li and Lee approach that takes into account dependence between the subgroups by modeling a common trend, but without imposing convergence to that common trend. The latter is important, as it is a strong assumption that educational group-specific trends converge, and the study of Van Baal showed that this was indeed not the case [18]. As compared to Van Baal et al., our study additionally includes mortality data from neighboring countries. This was also done in recent national projections in the Netherlands [2, 3] and Belgium [8], and has been shown to increase the robustness of the projections [5, 48]. We could make use of unique data on mortality by education from five European countries based on individual mortality follow-up, that were collected and harmonized by Erasmus MC and were used in prior publications [9, 28, 39]. As can be seen from Fig. 1, the trend in education-specific mortality for the five European countries (middle panels) is quite stable and the deviation of Dutch education-specific mortality from the international education-specific mortality (lower panels) is very small and shows no systematic pattern. This indeed suggests that by adding data from other European countries, we can produce more robust projections. In addition, as compared to the mortality projection by Van Baal et al., we included also younger ages (35 +) as well as more recent data, used a novel one-step estimation procedure proposed by Liu et al. [34] instead of the standard multi-step approach, used the Kannisto method to estimate and project mortality rates above age 85 [35], and estimated separate models for men and women.

A limitation of our study is that information on educational attainment was not available for the entire population (11 million out of 17.2 in million in 2018, but with lower coverage for older ages and early years). We applied correction factors additionally based on mortality data aggregated across all education groups and the group with missing education, following the approach developed by Statistics Netherlands for their calculations of life and health expectancy by education [26]. We did not include years prior to 2006 as these years were more impacted by this correction, and used mortality rates by education only up to age 85. Another limitation was that the time series of mortality by education were rather short for the Netherlands (2006–2018), particularly as compared to times series used in projections of the total population (by gender). Relying on short time series always yields the risk of extrapolating tendencies that are, in fact, only temporary. To mitigate this risk, we included longer time series of mortality for the total population (1970–2018) as well as longer time series of mortality by education from other North-Western European countries (1990–2018). The fact that deviations between average mortality in the Dutch education groups and average mortality in the international education groups are very small and behave like random noise (see bottom panels in Fig. 1) shows that the international education-specific trends are likely representative for the Dutch education-specific trends.

Another limitation is that our data did not allow us to take into account cohort effects in addition to period effects, nor did we make separate projections for smokers and non-smokers**.** This may have resulted in too optimistic mortality rates of women at older ages, particularly of low educated women. Based on the model of the smoking epidemic, we expect that current less favorable developments at middle ages in this group will shift to older ages as the affected cohorts get older. This would imply that also educational differences in remaining life expectancy at ages 35 and 65 could be larger than based on our projection.

Finally, our study provides best-estimate projections of future trends but does not quantify the corresponding uncertainty. The use of a three-layer Lee and Li model using datasets of different lengths and extrapolations to ages 85 + likely leads to considerable uncertainty. Moreover, the (unavoidable) choice of a specific projection model leads to so-called “model uncertainty”, which may be substantial and is not captured by prediction intervals [6]. According to Stoeldraijer [6] Statistics Netherlands takes 10 years as indication for the uncertainty of life expectancy at birth, 40 years ahead. We present results up to 2048, so maximum 30 years ahead, but our extrapolations are based on shorter time series and smaller numbers of observations because of stratification by education. We therefore expect large uncertainty intervals as well. Our outcomes should therefore be interpreted as extrapolations of past trends that give an indication of likely future developments if these trends continue, rather than a prognosis of future mortality and life expectancy.

### Comparison with recent trends and with other projections

The projected persistence and increase of inequalities in life expectancy between socioeconomic groups found in our study, is in line with the general picture arising from the existing literature on recent trends. For the Netherlands, inequalities in life expectancy by income quintile at age 40 increased between 2005 and 2015 [49]. For other European countries, there was also no evidence for a reduction in inequalities [43, 50–52]*.* Only for Norway the evidence was mixed, with one out of three studies showing a reduction in inequalities based on a comparison of three cohort studies [53], but the other studies pointing at persistent or increasing inequalities [53–55]. Longer time trends for the United Kingdom showed for men a widening socio-economic gap in life expectancy at birth during the 1980s and 1990s, but a reduction of the gap during the 2000s, and for women a widening gap since the mid-1990s. Trends in socioeconomic inequalities in life expectancy between areas in England point at a recent widening of inequalities for both genders. For women living in the most deprived areas in England there was even a reduction in life expectancy [56].

Regarding consistency with other projections, we are aware of only three projections using individual socioeconomic position, i.e., excluding studies that use area-based indicators: one for life expectancy at age 65 for the Netherlands using three education groups [18], one for life expectancy at birth for South Korea using income quantiles [16], and one for Denmark using an affluence index [17]. Similar to our study, the older Dutch projection by Van Baal et al. projected increasing life expectancy at age 65 for all educational groups for both men and women, with smaller increases for the low educated than for the high educated. In addition to projections by educational group, we are aware of a projection for South Korea, using income as socioeconomic status measure [16]. That study projects increasing inequalities between income quintiles in life expectancy at birth of the total population as well as for women, but for men the inequalities were projected to decrease. However, developments in South Korea may be less comparable to European countries. Moreover, using life expectancy at birth combined with income as socioeconomic status variable makes the outcomes less comparable to ours. For the United Kingdom there are also some studies that use area deprivation measures instead of individual socioeconomic measures [20, 57]. These studies project that inequalities in life expectancy between areas will further increase [20, 57]. In contrast, the study for Denmark [17] finds convergence of the lowest socioeconomic groups towards the other groups and constant inequalities for the other groups. This, however, may be due to the fact that their model imposes convergence of the group-specific trends to the common trend.

The scarcity of projections by education or other socioeconomic position measures may well reflect the fact that making mortality projections of different socioeconomic position groups is a more challenging and uncertain exercise than projections of the national population. Specific limitations related to the Dutch data add to this. Re-assuring, however, was that projected mortality rates for women were lower than for men in all education groups and that we did not project cross-overs between educational groups. Also re-assuring was that in line with prior evidence [58], we projected larger inequalities between the low and high educated for men than between high and low educated women. Our projection suggests decreasing gender gaps, which fits with known evidence on the impact of the smoking epidemic [30, 44]. Finally, our projections fit well with the projections made by Statistics Netherlands for the total population (by gender, but not by education) based on longer time series, finer-graduated data (single age and single calendar year) and avoiding uncertainty related to using mortality data by education. Statistics Netherlands projected life expectancy at age 65 to be 22.1 in 2048, which is consistent with our estimates of 19.7 years for low educated, 22.0 years for mid educated and 23.9 for the high educated [59]. For women, projected life expectancy by Statistics Netherlands at age 65 is 25.1 as compared to 23.0 for low, 25.9 for the mid and 26.1 for the high educated in our study.

## Conclusion and implications

Based on our projections, we conclude that whereas all educational groups are expected to experience further increases in life expectancy between age 35 and 85 as well as in remaining life expectancy at age 35 and 65, low educated are not expected to catch up with higher educated peers. Trends for low educated women are expected to be the least favorable, particularly between ages 35 and 85. We further conclude that inequality in mortality and life expectancy between educational groups are persistent and may increase further in the future.

This persistence of inequalities in life expectancies between education groups combined with the declining share of lower educated and increasing share of higher educated groups in the population, has implications for the interpretation of future changes in period life expectancy of national populations and for their use in economic and social policy. First, part of the increase in period life expectancy does not reflect a reduction in mortality rates of the different groups, but an increase in the fraction of high educated in the population. Second and more importantly, national projections mask the large and persistent inequalities between the socioeconomic groups we found in our study. This is particularly relevant when national projections of life expectancy are used in pension policies, as is the case in many countries. In the Netherlands, for example, pension age is linked to projected changes in life expectancy of the national population [60]. This causes equity concerns. To assess the viability of such policies, there is a need to address variations in level and future trends in life expectancy of different socioeconomic groups. In addition, variations in years lived in good health relative to the increased pension age [38] are also important to consider in a pension reform, as poor health may push people to unemployment or disability benefits before they reach the retirement age. Differentiation of the statutory pension age by socio-economic position is a possible scenario to address both inequalities in life expectancy after retirement as well as inequalities in the expected number of years in good health before the retirement. However, practical implementation of a differentiated pension age is highly complicated and politically contentious. It involves dividing people in groups based on criteria that are partly subjective, while the financial consequences of the division can be substantial.

## Supplementary Information

**Additional file 1: Appendix 1.** European data by education and gender.

**Additional file 2: Appendix 2.**  Model and estimation procedure.

**Additional file 3: Appendix 3.** Life expectancy (LE) between age 35 and 80. 

**Additional file 4: Appendix 4.** Life expectancies by calendar year.

**Additional file 5: Appendix 5.** Life expectancy between age 35 and 85 based on observed and modelled rates.
