# Supplementary material for: Future trends of life expectancy by education in the Netherlands
Source: BMC Public Health. 2022 Sep 2;22:1664. doi: 10.1186/s12889-022-13275-w (PMC9438160; doi:10.1186/s12889-022-13275-w)
Supplement: Supplementary file 1 — Additional file 1: Appendix 1. European data by education and gender. [file 12889_2022_13275_MOESM1_ESM.pdf]

Supplementary material to "Future trends of life expectancy by education in the Netherlands"

by WJ Nusselder & AMB De Waegenaere et al, BMC Public Health 2022,

Appendix 1 European data by education and gender

Table A1-1 European mortality data by sex, age, education

Country	Type	Years	Census date	Length of follow-up (year)	Geographic coverage
Belgium	Longitudinal	March 1 1990 – Dec 31, 1997	1.3.1997	6 Y, 10 M	National
		Oct, 1 2001 – Dec 31, 2011	1.19.2001	10, 3 M	National
Denmark	Longitudinal	Jan 1, 1995- Dec 31 1999	1995	5	National
		Jan 1, 2000- Dec 31 2004	2000	5	National
		Jan 1, 2005- Dec 31 2009	2005	5	National
		Jan 1 2010- 3Dec 31 2014	2010	5	National
		March 2011-December 2013	March 2011	2 Y, 10 M	National
Finland	Longitudinal	Dec 31, 1990 – Dec 31, 2000	31.12.1990	10	National
		Dec 31, 2000 – Dec 31, 2010	31.12.2000	10	National
		Dec 31, 2010 –.Dec 31, 2014	31.12.2010	4	National
Norway	Longitudinal	Nov, 1990 – Dec, 2001	Nov, 1990	11	National
		Nov, 2001 – Dec, 2009	Nov, 2001	8	National
		Jan 2000 – Dec 2008	1.2001	9	National
Switzerland	Longitudinal	Dec 4, 1990 – Dec 5, 2000	4.12.1990	10	National
		Dec 31, 2000 – Dec 31, 2010	5.12.2000	10	National
		Dec 31 2010 – Dec 31, 2014	31.12.2010	4	National

Table A1-2 Summary of total number of deaths and person years, based on European

Country	Education	Deaths	Person Years
Belgium	Low	1165334	51038290
Belgium	Mid	177225	21504660
Belgium	High	118836	18624384
Denmark	Low	393680	20345764
Denmark	Mid	228399	25201252
Denmark	High	85028	15028869
Finland	Low	1587379	62060261
Finland	Mid	255404	30618591
Finland	High	168860	24107525
Netherlands	Low	115021	14912715
Netherlands	Mid	72196	21782186
Netherlands	High	37231	20698799
Norway	Low	992196	29689344
Norway	Mid	477520	25580187
Norway	High	96907	8631005
Switzerland	Low	587125	20831871
Switzerland	Mid	478580	41367716
Switzerland	High	119882	14022826
