# Supplementary material for: Future trends of life expectancy by education in the Netherlands
Source: BMC Public Health. 2022 Sep 2;22:1664. doi: 10.1186/s12889-022-13275-w (PMC9438160; doi:10.1186/s12889-022-13275-w)
Supplement: Supplementary file 2 — Additional file 2: Appendix 2. Model and estimation procedure. [file 12889_2022_13275_MOESM2_ESM.pdf]

Supplementary material to "Future trends of life expectancy by education in the Netherlands" by WJ Nusselder & AMB De Waegenaere et al, BMC Public Health 2022

Appendix 2: Model and estimation procedure

1. The model

To model Dutch-education-specific mortality rates, we use a three-layer Li and Lee (2005) model. Each layer is modeled as a Lee and Carter (1992) model with identification constraints as in Liu et al. (2019). Below we present the model for the three layers.

We denote x for an age group, t for a time period, $g \in \{m, f\}$ for gender, in which m (f) stands for male (female), and $e \in \{l, m, h\}$ for educational level, in which l (m, and h) stands for low (medium, and high) education.

Layer 1: Aggregate international mortality

In the first layer, we model international mortality rates for males and females separately. Specifically, let $m^g_{x,t}$ be the central mortality rate for gender $g \in \{m, f\}$, age group x, and year t for a group of countries. Then:

$$\ln\ m^g_{x,t} = A^{(1),g}_x + B^{(1),g}_x K^{(1),g}_t + u^{(1),g}_{x,t}, \tag{1}$$

where $u^{(1),g}_{x,t}$ is a measurement error with zero mean and finite variance. The identification constraints are:

$$\sum_x A^{(1),g}_x = 0 \ \text{ and } \ \sum_x B^{(1),g}_x = 1, \qquad \text{for } \ g \in \{m, f\}. \tag{2}$$

Layer 2: International education-specific deviation

In the second layer, we model the difference between international education-specific logarithm central death rates and international logarithm central death rates from layer 1.

Specifically, let $m_{x,t}^{g,e}$ be the central mortality rate for gender $g \in \{m, f\}$, age group x, educational level e, and year t for the group of countries from layer 1. Then:

$$\ln\ m_{x,t}^{g,e} - \ln\ m_{x,t}^{g} = A_x^{(2),g,e} + B_x^{(2),g,e} K_t^{(2),g,e} + u_{x,t}^{(2),g,e}, \quad (3)$$

where $u_{x,t}^{(2),g,e}$ is a measurement error with zero mean and finite variance. The identification constraints are:

$$\sum_x A_x^{(2),g,e} = 0,\ \text{and}\ \sum_x B_x^{(2),g,e} = 1. \quad (4)$$

Layer 3: Dutch education-specific deviation

In the third layer, we model the difference between the Dutch education-specific log central death rate and the international education-specific log central death rate from layer 2. Specifically, let $m_{x,t}^{g,e,NL}$ be the central mortality rate for the Netherlands for gender $g \in \{m, f\}$, age group x, educational level e and year t. Then:

$$\ln\ m_{x,t}^{g,e,NL} - \ln\ m_{x,t}^{g,e} = A_x^{(3),g,e,NL} + B_x^{(3),g,e,NL} K_t^{(3),g,e,NL} + u_{x,t}^{(3),g,e,NL}, \quad (5)$$

where $u_{x,t}^{(3),g,e,NL}$ is a measurement error with zero mean and finite variance. The identification constraints are:

$$\sum_x A_x^{(3),g,e,NL} = 0 \text{ and } \sum_x B_x^{(3),g,e,NL} = 1. \quad (6)$$

Dutch education-specific mortality

Dutch education-specific log mortality ($\ln\ m_{x,t}^{g,e,NL}$) is the sum of the three layers. Com-

bining (1), (3), and (5) yields:

$$\begin{aligned}
\ln m_{x,t}^{g,e,NL} &= \ln\ m_{x,t}^{g} + (\ln\ m_{x,t}^{g,e} - \ln\ m_{x,t}^{g}) + (\ln\ m_{x,t}^{g,e,NL} - \ln\ m_{x,t}^{g,e}), \\
&= A_x^{(1),g} + A_x^{(2),g,e} + A_x^{(3),g,e,NL} + B_x^{(1),g} K_t^{(1),g} + B_x^{(2),g,e} K_t^{(2),g,e} + B_x^{(3),g,e,NL} K_t^{(3),g,e,NL} \\
&\qquad + u_{x,t}^{(1),g} + u_{x,t}^{(2),g,e} + u_{x,t}^{(3),g,e,NL}. \qquad (7)
\end{aligned}$$

2. The data

We use the following data to estimate the model:

- Layer 1: number of deaths and exposures for Finland, Norway, Denmark, Belgium, Switzerland, and the Netherlands, by gender, by five year age group for the age range 35-39 up to 80-84, and by single calendar year for the years 1970 till 2016. Data are obtained from the Human Mortality Database (HMD)[24].

- Layer 2: number of deaths and exposures by gender and educational level for Finland, Norway, Denmark, Belgium, Switzerland, and the Netherlands, by five year age group for the age range 35-39 up to 80-84. The data is by seven, five, two or single calendar years depending on the country. The data was collected and harmonized as part the LIFEPATH and earlier European projects at Erasmus MC. To use the data in the estimation procedure, we have allocated each year range to a midpoint year. To ensure that for each midpoint year we can use data for at least four of the six countries, we have included in our analysis only the midpoint years 1993, 1998, 2003, 2008, and 2013. In case a calculated midpoint deviated at most one year from one of these included midpoint years, we allocated the data to the closest included midpoint. The left panel in Table 1 displays the calculated midpoints; the right panel displays the allocated midpoints. The table shows that the shifts between a calculated and an allocated midpoint year were maximum one year.

- Layer 3: number of deaths and exposures in the Netherlands by gender, by educa-

Country	Calculated midpoint					Allocated midpoint				
Belgium	1994		2004	2009		1993		2003	2008	
Denmark		1997	2002	2007	2012		1998	2003	2008	2013
Finland	1993	1998	2003	2008	2013	1993	1998	2003	2008	2013
Netherlands				2008	2013				2008	2013
Norway	1993	1998	2004	2008		1993	1998	2003	2008	
Switzerland	1993	1998	2003	2008	2013	1993	1998	2003	2008	2013

Table 1: Left panel: calculated midpoint years. Right panel: allocated midpoint years. A blue entry in the left panel indicates that the midpoint year was shifted by one year.

tional level, by five year age group for the age range 35-39 up to 80-84, and by single calendar year for the years 2006, 2007, ..., 2018. Data were obtained through individual data linkage of register data of all persons living in the Netherlands, within the secure environment of Statistical Netherlands.

3. Parameter estimation

In this section we describe the estimation procedure, taking into account the different time periods for which data is used in the three layers. An overview of the data is provided in Table 2.

	Age range	Year range
HMD	35-39,40-44,...,80-84	1970,1971,...,2017,2016
INT EDU	35-39,40-44,...,80-84	1993, 1998, 2003, 2008, 2013
NL EDU	35-39,40-44,...,80-84	2006,2007,...,2017,2018

Table 2: Overview of the data

3.1 The central mortality rates

We denote x for an age group, t for a time period, $g \in \{m, f\}$ for gender, and $e \in \{l, m, h\}$ for the educational level, and $c \in C := \{FL, NW, DM, BE, SWZ, NL\}$ for the country. Moreover, we use the following notation:

- $D^{g,c}_{x,t}$: the number of deaths in period t for gender g and age group x in country $c \in C$;
- $E^{g,c}_{x,t}$: the exposure to death in period t for gender g, age group x in country $c \in C$;

- $D^{g,e,c}_{x,t}$: the number of deaths allocated to midpoint year t for gender g, age group x, and educational level e in country $c \in C$;
- $E^{g,e,c}_{x,t}$: the exposure to death allocated to midpoint year t for gender g, age group x, and educational level e in country $c \in C$.

Then, for genders $g \in \{m, f\}$, age groups $x \in \{35-39, 40-44, \ldots, 80-84\}$, educational levels $e \in \{l, m, h\}$, and years t, the central death rates at the three levels are estimated as follows:

$$m^{g}_{x,t} = \textstyle\sum_{c\in C_t} D^{g,c}_{x,t} / \sum_{c\in C_t} E^{g,c}_{x,t}, \text{ for } t \in \{1970, 1971, \ldots, 2016\}; \tag{8}$$

$$m^{g,e}_{x,t} = \textstyle\sum_{c\in C_t} D^{g,e,c}_{x,t} / \sum_{c\in C_t} E^{g,e,c}_{x,t}, \text{ for } t \in \{1993, 1998, 2003, 2008, 2013\}; \tag{9}$$

$$m^{g,e,NL}_{x,t} = D^{g,e,NL}_{x,t} / E^{g,e,NL}_{x,t}, \text{ for } t \in \{2006, 2007, \ldots, 2018\}, \tag{10}$$

where C_t is the set of countries with data allocated to midpoint year t, as displayed in Table 1.

3.2 Choice of time series processes in the three layers

We use the modified estimation of Liu et al. (2019). An important difference between this estimation and the traditional Singular-Value-Decomposition (SVD) estimation (Lee and Carter 1992) is that estimation of the parameters $A_{x,t}$ and $B_{x,t}$ depends on the specification of the time series process for the time varying component K_t. Hence, the selection of time series processes for the three layers is the first step in the estimation procedure.

In this section we discuss the selection of a time series process for $K^{(1),g}_t$,$K^{(2),g,e}_t$, and $K^{(3),g,e,NL}_t$ for $g \in \{m, f\}$ and $e \in \{l, m, h\}$. In each case, we make a choice between a Random Walk with Drift (RWD) and AutoRegressive process of order 1 (AR1).

Let us denote:

$$\begin{aligned} Z_t^{(1),g} &= \sum_x \ln(m_{x,t}^g), \\ Z_t^{(2),g,e} &= \sum_x \left(\ln\ m_{x,t}^{g,e} - \ln\ m_{x,t}^g\right), \\ Z_t^{(3),g,e,NL} &= \sum_x \left(\ln\ m_{x,t}^{g,e,NL} - \ln\ m_{x,t}^{g,e}\right). \end{aligned} \tag{11}$$

It then follows from the identification constraints that:

$$\begin{aligned} K_t^{(1),g} &= Z_t^{(1),g} - \bar{u}_t^{(1),g}, \\ K_t^{(2),g,e} &= Z_t^{(2),g,e} - \bar{u}_t^{(2),g,e}, \\ K_t^{(3),g,e,NL} &= Z_t^{(3),g,e,NL} - \bar{u}_t^{(3),g,e,NL}, \end{aligned} \tag{12}$$

where $\bar{u}_t^{(1),g} = \sum_x u_{x,t}^{(1),g}$, $\bar{u}_t^{(2),g,e} = \sum_x u_{x,t}^{(2),g,e}$, and $\bar{u}_t^{(3),g,e,NL} = \sum_x u_{x,t}^{(3),g,e,NL}$.
Figure 1 displays $Z_t^{(1),g} = \sum_x \ln(m_{x,t}^g)$ (black circles), as well as $Z_t^{(1),g}+Z_t^{(2),g,e} = \sum_x \ln(m_{x,t}^{g,e})$ (red circles) and $Z_t^{(1),g} + Z_t^{(2),g,e} + Z_t^{(3),g,e} = \sum_x \ln(m_{x,t}^{g,e,NL})$ (blue dots) for low (left panels) medium (middle panels) and high (right panels) educational level. The top (bottom) panels correspond to males (females).

- Selecting a process for $K_t^{(1),g}$ (international mortality): Figure 1 shows that for both males and females, $Z_t^{(1),g}$ follows a relatively stable declining trend. Hence, we choose a RWD process for $K_t^{(1),g}$ for both genders:

  $$K_t^{(1),g} = \mu^{(1),g} + K_{t-1}^{(1),g} + \epsilon_t^{(1),g}, \tag{13}$$

  where $\mu^{(1),g}$ is the drift term and $\epsilon_t^{(1),g}$ is an innovation with zero mean and finite variance.[1]

- Selecting a process for $K_t^{(2),g,e}$ (international education-specific deviation): Comparing the black circles and red circles suggests that for the low educated, the trend

[1] We do not specify the distribution of the measurement errors and the innovations because we focus on point estimates.

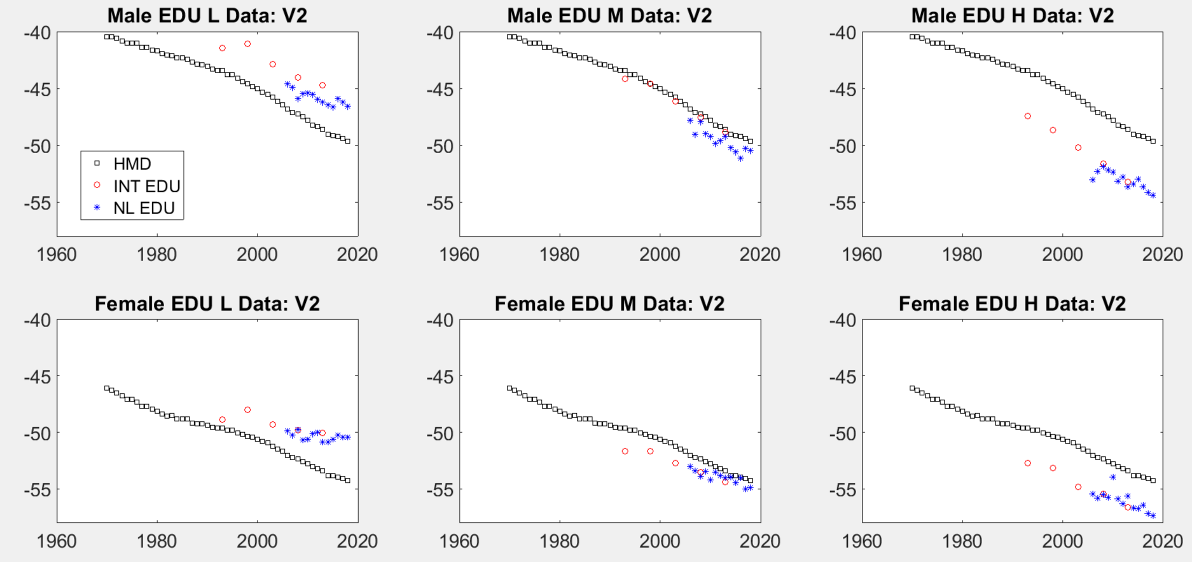

Figure 1: The figure displays $\sum_x \ln(m^g_{x,t})$ (black circles), as well as $= \sum_x \ln(m^{g,e}_{x,t})$ (red circles) and $\sum_x \ln(m^{g,e,NL}_{x,t})$ (blue dots) for low (left panels) medium (middle panels) and high (right panels) educational level. The top (bottom) panels correspond to males (females).

diverges from the overall population trend whereas for the middle educated, the trend seems to be parallel. Hence, we choose a RWD for the low education group and an AR1 process for the middle education group. For the high educational group, Figure 1 does not yield a clear conclusion. We have therefore estimated the time process both for the case of an RWD process and an AR1 process. Because the differences were negligible, we present results for the case where $K^{(2),g,h}_t$ follows a RWD. hence:

$$K^{(2),g,e}_t = \mu^{(2),g,e} + K^{(2),g,e}_{t-1} + \epsilon^{(2),g,e}_t, \qquad \text{for } e = l, h, \tag{14}$$

$$K^{(2),g,m}_t = \phi^{(2),g,m}_0 + \phi^{(2),g,m}_1 K^{(2),g,e}_{t-1} + \epsilon^{(2),g,m}_t, \tag{15}$$

where $\epsilon^{(2),g,e}_t$ are innovations with mean zero and finite variance.

- Selecting a process for $K^{(3),g,e,NL}_t$ (Dutch education-specific deviation): Figure 1 suggests that the deviation between international education-specific mortality and

Dutch education-specific mortality fluctuates around a (small) constant. Therefore, we select an ARIMA(0,0,0) process with constant for this deviation:

$$K_t^{(3),g,e,NL} = c^{(3),g,e,NL} + u_t^{(3),g,e,NL},, \qquad \text{for } e = l, m, h, \tag{16}$$

where $u_t^{(3),g,e,NL} \sim N(0, \sigma^2_{g,e})$.

The selected time series processes for the second and third layer are summarized in Table 3. The selection is the same for males and females.

	Low	Medium	High
$K_t^{(2),g,e}$	RWD	AR1	RWD
$K_t^{(3),g,e,NL}$	ARIMA(0,0,0) with constant	ARIMA(0,0,0) with constant	ARIMA(0,0,0) with constant

Table 3: Time series processes for $K_t^{(2),g,e}$ and $K_t^{(3),g,e,NL}$

3.3 Parameter estimation

In each layer we use the estimation procedure described in Liu et al. (2019) combined with interpolation/extrapolation to account for the fact that the data for the three layers has different levels of granularity. Specifically, the data for the HMD population is available for $t \in \{1970, 1971, \ldots, 2016\}$, international education-specific mortality is available only for $t \in \{1993, 1998, 2003, 2008, 2013\}$, and international education-specific mortality is available for $t \in \{2006, 2007, \ldots, 2018\}$. Therefore, in the first and second layer we first estimate the model parameters with the available data, using the estimation procedure described in Liu et al. (2019). We then use extrapolation to extend the HMD data till 2018 in layer 1 and we use interpolation and extrapolation to create yearly data for the period 2006 until 2018 in layer 2.

First layer

For $K_t^{(1),g}$, we selected a RWD process, i.e., $K_t^{(1),g} = \mu^{(1),g} + K_{t-1}^{(1),g} + \epsilon_t^{(1),g}$. The trend is estimated using international mortality data for the years for $t \in \{1970, 1971, \ldots, 2016\}$:

$$\hat{\mu}^{(1),g} = \arg\min_{\mu^{(1),g}} \sum_{t=1971}^{2016} \left[Z_t^{(1),g} - \mu^{(1),g} - Z_{t-1}^{(1),g} \right]^2 = \frac{Z_{2016}^{(1),g} - Z_{1970}^{(1),g}}{46}. \quad (17)$$

Then, $\hat{A}_x^{(1),g}$ and $\hat{B}_x^{(1),g}$ are obtained by solving the least squares problem:

$$\min_{A_x^{(1),g}, B_x^{(1),g}} \sum_x \sum_{t=1970}^{2016} \left[\ln(m_{x,t}^g) - A_x^{(1),g} - B_x^{(1),g} Z_t^{(1),g} \right]^2, \quad (18)$$

This yields (see Liu et al. (2019)):

$$\hat{A}_x^{(1),g} = \frac{\sum_t \ln(m_{x,t}^g) \sum_t [Z_t^{(1),g}]^2 - \sum_t \ln(m_{x,t}^g) Z_t^{(1),g} \sum_t Z_t^{(1),g}}{47 \sum_t [Z_t^{(1),g}]^2 - (\sum_t Z_t^{(1),g})^2}, \quad (19)$$

$$\hat{B}_x^{(1),g} = \frac{47 \sum_t \ln(m_{x,t}^g) Z_t^{(1),g} - \sum_{t=1}^T \ln(m_{x,t}^g) \sum_t Z_t^{(1),g}}{47 \sum_t [Z_t^{(1),g}]^2 - (\sum_t Z_t^{(1),g})^2}. \quad (20)$$

Because Dutch education-specific data is available also for $t = 2017$ and $t = 2018$, we generate "pseudo" data for these years for international mortality using the estimated parameters and the extrapolated trend, i.e., for $t \in \{2017, 2018\}$:

$$\ln\ m_{x,t}^g = \hat{A}_x^{(1),g} + \hat{B}_x^{(1),g} \hat{K}_t^{(1),g} = \hat{A}_x^{(1),g} + \hat{B}_x^{(1),g} \left[Z_{2016}^{(1),g} + (t - 2016) \hat{\mu}^{(1),g} \right]. \quad (21)$$

Second layer

We first estimate the model parameters for each educational level in layer 2 using data for years 1993, 1998, 2003, 2008, and 2013. We then use the estimated parameters to interpolate and extrapolate the data to the range $t \in \{1993, 1994, \ldots, 2018\}$.

Low and high education: we selected an RWD process for $K_t^{(2),g,e}$, i.e., $K_t^{(2),g,e} =$

$\mu^{(2),g,e} + K_{t-1}^{(2),g,e} + \epsilon_t^{(1),g,e}$. Because international education-specific mortality data is used only for $t \in \{1993, 1998, 2003, 2008, 2013\}$, we let $t_j = 1993 + 5(j-1)$ for $j = 1, 2, \ldots 5$. Then, the least squares estimator $\hat{\mu}^{(2),g,e}$ is given by:

$$\hat{\mu}^{(2),g,e} = \underset{\mu^{(2),g,e}}{\arg\min} \sum_{j=2}^{5} \left[Z_{t_j}^{(2),g,e} - 5\mu^{(2),g,e} - Z_{t_{j-1}}^{(2),g,e} \right]^2 = \frac{Z_{2013}^{(2),g,e} - Z_{1993}^{(2),g,e}}{20}. \tag{22}$$

Then, $\hat{A}_x^{(2),g,e}$ and $\hat{B}_x^{(2),g,e}$ are obtained by solving the least squares problem:

$$\min_{A_x^{(2),g,e}, B_x^{(2),g,e}} \sum_{j=1}^{5} \sum_x [\ln(m_{x,t_j}^{g,e}) - \ln(m_{x,t_j}^{g}) - A_x^{(2),g,e} - B_x^{(2),g,e} Z_{t_j}^{(2),g}]^2. \tag{23}$$

Hence, $\hat{A}_x^{(2),g,e}$ and $\hat{B}_x^{(2),g,e}$ are as in (19) and (20), with $t \in \{1970, 1971, \ldots, 2016\}$ replaced by $t_j \in \{1993, 1998, 2003, 2008, 2013\}$ and $\ln(m_{x,t}^g)$ replaced by $\ln(m_{x,t_j}^{g,e}) - \ln(m_{x,t_j}^g)$.

Middle education: we selected an ARIMA(1,0,0) process, i.e.,: $K_t^{(2),g,m} = \phi_0^{(2),g,m} + \phi_1^{(2),g,m} K_{t-1}^{(2),g,m} + \epsilon_t^{(2),g,m}$, where $\epsilon_t^{(2),g,m}$ are innovations with mean zero and finite variance. Again let $t_j = 1993 + 5(j-1)$ for $j = 1, 2, \ldots 5$. Then, it holds that:

$$K_{t_j}^{(2),g,m} = \varphi_0^{(2),g,m} + \varphi_1^{(2),g,m} K_{t_{j-1}}^{(2),g,e} + \tilde{\epsilon}^{(2),g,m}, \text{ for } j = 2, 3, 4, 5, \tag{24}$$

where

$$\varphi_0^{(2),g,m} = \phi_0^{(2),g,m} \left[1 + \phi_1^{(2),g,m} + \left(\phi_1^{(2),g,m}\right)^2 + \left(\phi_1^{(2),g,m}\right)^3 + \left(\phi_1^{(2),g,m}\right)^4 \right], \tag{25}$$

$$\varphi_1^{(2),g,m} = \left(\phi_1^{(2),g,m}\right)^5. \tag{26}$$

The least-square problem for the parameters in (24) for $e = m$ is:

$$\min_{\varphi_0^{(2),g,e}, \varphi_1^{(2),g,e}} \sum_{j=2}^{5} \left[Z_{t_j}^{(2),g,e} - \varphi_0^{(2),g,e} - \varphi_1^{(2),g,e} Z_{t_{j-1}}^{(2),g,e} \right]^2, \tag{27}$$

We use the bias-corrected estimation in Liu et al. (2019), which is an instrumental variable (IV) regression with the lagged variable $Z^{(2),g,e}_{t_{j-2}}$ as an instrument for $Z^{(2),g,e}_{t_{j-1}}$. This IV regression addresses potential endogeneity problems that arise if the independent variable is correlated with the innovation term. This yields for $e = m$ (see Liu et al. (2019)):

$$\hat{\varphi}^{(2),g,e}_0 = \frac{\sum_{s=3}^5 Z^{(2),g,e}_{t_s} \sum_{j=3}^5 [Z^{(2),g,e}_{t_{j-1}} Z^{(2),g,e}_{t_{j-2}}] - \sum_{s=3}^5 Z^{(2),g,e}_{t_{s-1}} \sum_{j=3}^5 [Z^{(2),g,e}_{t_j} Z^{(2),g,e}_{t_{j-2}}]}{(5-2)\sum_{j=3}^5 [Z^{(2),g,e}_{t_{j-1}} Z^{(2),g,e}_{t_{j-2}}] - \sum_{s=3}^5 Z^{(2),g,e}_{t_{s-1}} \sum_{j=3}^5 Z^{(2),g,e}_{t_{j-2}}},$$

$$\hat{\varphi}^{(2),g,e}_1 = \frac{(5-2)\sum_{s=3}^5 [Z^{(2),g,e}_{t_s} Z^{(2),g,e}_{t_{s-2}}] - \sum_{s=3}^5 Z^{(2),g,e}_{t_s} \sum_{j=3}^5 Z^{(2),g,e}_{t_{j-2}}}{(5-2)\sum_{s=3}^5 [Z^{(2),g,e}_{t_{s-1}} Z^{(2),g,e}_{t_{s-2}}] - \sum_{s=3}^5 Z^{(2),g,e}_{t_{s-1}} \sum_{j=3}^5 Z^{(2),g,e}_{t_{j-2}}}.$$

The corresponding parameter estimates $\hat{\phi}^{(2),g,e}_0$ and $\hat{\phi}^{(2),g,e}_1$ for $e = m$ now follow from (25) and (26), i.e.,:

$$\hat{\phi}^{(2),g,m}_1 = \left(\hat{\varphi}^{(2),g,m}_1\right)^{\frac{1}{5}}, \quad (28)$$

$$\hat{\phi}^{(2),g,m}_0 = \hat{\varphi}^{(2),g,m}_0 / \left[1 + \hat{\varphi}^{(2),g,m}_1 + \left(\hat{\varphi}^{(2),g,m}_1\right)^2 + \left(\hat{\varphi}^{(2),g,m}_1\right)^3 + \left(\hat{\varphi}^{(2),g,m}_1\right)^4\right]. \quad (29)$$

Moreover, the estimates of $A^{(2),g,e}_x$ and $B^{(2),g,e}_x$ for $e = m$ are given by:

$$\hat{A}^{(2),g,e}_x = \frac{\sum_{s=3}^5 \Delta \ln\ m^{g,e}_{x,t_s} \sum_{j=3}^5 [Z^{(2),g,e}_{t_j} Z^{(2),g,e}_{t_{j-1}}] - \sum_{s=3}^5 \Delta \ln\ m^{g,e}_{x,t_s} Z^{(2),g,e}_{t_{s-1}} \sum_{j=3}^5 Z^{(2),g,e}_{t_j}}{(5-2)\sum_{j=3}^5 [Z^{(2),g,e}_{t_j} Z^{(2),g,e}_{t_{j-1}}] - \sum_{s=3}^5 Z^{(2),g,e}_{t_s} \sum_{j=3}^5 Z^{(2),g,e}_{t_{j-1}}},$$

$$\hat{B}^{(2),g,e}_x = \frac{(5-2)\sum_{s=3}^5 \Delta \ln\ m^{g,e}_{x,t_s} Z^{(2),g,e}_{t_{s-1}} - \sum_{s=3}^5 \Delta \ln\ m^{g,e}_{x,t_s} \sum_{j=3}^5 Z^{(2),g,e}_{t_{j-1}}}{(5-2)\sum_{j=3}^5 [Z^{(2),g,e}_{t_j} Z^{(2),g,e}_{t_{j-1}}] - \sum_{s=3}^5 Z^{(2),g,e}_{t_s} \sum_{j=3}^5 Z^{(2),g,e}_{t_{j-1}}},$$

where $\Delta \ln\ m^{g,e}_{x,t_s} = \ln(m^{g,e}_{x,t}) - \ln(m^{g}_{x,t})$.

Interpolation and extrapolation of $Z^{(2),g,e}_t$. Because we have estimated the parameters of layer 2 based on international education-specific data for $t_j \in \{1993, 1998, 2003, 2008, 2013\}$ but Dutch education-specific data is available on one-year basis for the years $t \in \{2006, 2007, \ldots, 2018\}$,

we generate "pseudo" data for international mortality in years $t \in \{2006, 2007\} \cup \{2009, \ldots, 2012\} \cup \{2014, \ldots, 2018\}$ using the estimated parameters and the interpolated/extrapolated trend. Specifically, for $t \in \{2006, 2007\} \cup \{2009, \ldots, 2012\} \cup \{2014, \ldots, 2018\}$ we let:

$$\ln\ m_{x,t}^{g,e} = \hat{A}_x^{(2),g,e} + \hat{B}_x^{(2),g,e} \hat{K}_t^{(2),g,e}, \tag{30}$$

where $\hat{K}_t^{(2),g,e} = Z_t^{(2),g,e}$ for $t \in \{2003, 2008, 2013\}$, and:

$$\hat{K}_t^{(2),g,e} = \hat{\mu}^{(2),g,e} + \hat{K}_{t-1}^{(2),g,e}, \qquad \text{for } e \in \{l, h\}, \tag{31}$$

$$\hat{K}_t^{(2),g,m} = \hat{\phi}_0^{(2),g,m} + \hat{\phi}_1^{(2),g,m} \hat{K}_{t-1}^{(2),g,m}, \tag{32}$$

for $t \in \{2004, \ldots, 2007\} \cup \{2009, \ldots, 2012\} \cup \{2014, \ldots, 2018\}$.

Third layer

For $K_t^{(3),g,e,NL}$, we selected an ARIMA(0,0,0) process with a constant, i.e., $K_t^{(3),g,e,NL} = c^{(3),g,e,NL} + u_t^{(3),g,e,NL}$, where $u_t^{(3),g,e,NL}$ follows a normal distribution with zero mean and finite variance $\sigma^2_{(3),g,e}$. The constant $c^{(3),g,e,NL}$ can be estimated by:

$$\hat{c}^{(3),g,e,NL} = \frac{\sum_{t=2006}^{2018} Z_t^{(3),g,e,NL}}{13}.$$

Moreover, $\hat{A}_x^{(3),g,e,NL}$ and $\hat{B}_x^{(3),g,e,NL}$ are as in (19) and (20), with $t \in \{1970, 1971, \ldots, 2016\}$ replaced by $t \in \{2006, \ldots, 2018\}$ and $\ln(m_{x,t}^{g})$ replaced by $\ln(m_{x,t}^{g,e,NL}) - \ln(m_{x,t}^{g,e})$.

4. Projection

Best estimate projections for Dutch education-specific log mortality ($\ln\ m_{x,t}^{g,e,NL}$) by gender for $x \in \{35-39, 40-44, \ldots, 80-84\}$ and future years $t = 2019, 2020, \ldots$ are obtained by

combining (1), (3), and (5) and substituting in the parameter estimates. This yields:

$$\begin{aligned}\ln \hat{m}_{x,t}^{g,e,NL} &= \hat{A}_x^{(1),g} + \hat{A}_x^{(2),g,e} + \hat{A}_x^{(3),g,e,NL} \\ &\quad + \hat{B}_x^{(1),g}\hat{K}_t^{(1),g} + \hat{B}_x^{(2),g,e}\hat{K}_t^{(2),g,e} + B_x^{(3),g,e,NL}\hat{K}_t^{(3),g,e,NL},\end{aligned} \tag{33}$$

where

$$\hat{K}_t^{(1),g} = \hat{\mu}^{(1),g} + \hat{K}_{t-1}^{(1),g}, \tag{34}$$

$$\hat{K}_t^{(2),g,e} = \hat{\mu}^{(2),g,e} + \hat{K}_{t-1}^{(2),g,e}, \qquad \text{for } e = l, h, \tag{35}$$

$$\hat{K}_t^{(2),g,m} = \hat{\phi}_0^{(2),g,m} + \hat{\phi}_1^{(2),g,m} K_{t-1}^{(2),g,e}, \tag{36}$$

$$\hat{K}_t^{(3),g,e,NL} = \hat{c}^{(3),g,e,NL}, \tag{37}$$

with $\hat{K}_{2018}^{(1),g} = Z_{2018}^{(1),g}$, $\hat{K}_{2018}^{(2),g,e} = Z_{2018}^{(2),g,e}$, and $\hat{K}_{2018}^{(3),g,e,NL} = Z_{2018}^{(3),g,e,NL}$.

5. Old age extrapolation

The model and its parameters are estimated for age groups $x \in \{35{-}39, 40{-}44, \ldots, 80{-}84\}$. To extrapolate central mortality rates to age groups $x \in \{85-89, ..., 105-109\}$, we rely on the extrapolation that was implemented in the HMD for Dutch central death rates. The HMD provides central death rates for ages 0, 1-4, 5-9, ..., 75-79, 80-84, ..., 105-109, 110+ and years 1850, 1851, 1852, ..., 2006, 2007, ..., 2017, 2018, where central death rates for age groups above 80-84 are extrapolated using Kannisto.[2] For each gender and each age group $x \in \{80-84, ..., 105-109\}$, we let $m_{x,t}^{g,NL}$ be the central death rate from HMD for the Netherlands. Moreover, for each age group x, we define x_{-1} as the age group just below x,

[2]We obtain the 5×1 life table of both genders of the Netherlands from HMD `https://www.mortality.org/hmd/NLD/STATS/mltper_5x1.txt` for male and the one with 'fltper' is for female. According to HMD protocol v6, death rates of ages above 80 are smoothed using the Kannisto method.

e.g., for $x = 85 - 89$, it holds that $x_{-1} = 80 - 84$. Then, we let

$$\Delta^g_{x,t} = \ln(\frac{m^{g,NL}_{x,t}}{1 - m^{g,NL}_{x,t}}) - \ln(\frac{m^{g,NL}_{x_{-1},t}}{1 - m_{x^{g,NL}_{-1},t}}),$$

for age groups $x \in \{85 - 89, ..., 105 - 109\}$ and years $t \in \{2006, 2007, ..., 2017, 2018\}$.

Now, Dutch education-specific mortality rates for gender g and years $t \in \{2006, 2007, ..., 2017, 2018\}$ are extrapolated to age groups $x \in \{85 - 89, ..., 105 - 109\}$ as follows:

$$\ln(\frac{m^{g,e,NL}_{x,t}}{1 - m^{g,e,NL}_{x,t}}) - \ln(\frac{m^{g,e,NL}_{x_{-1},t}}{1 - m^{g,e,NL}_{x_{-1},t}}) = \Delta^g_{x,t}. \tag{38}$$

References

Lee, R. D. and L. R. Carter (1992). Modeling and forecasting us mortality. *Journal of the American statistical association 87*(419), 659–671.

Liu, Q., C. Ling, D. Li, and L. Peng (2019). Bias-corrected inference for a modified lee–carter mortality model. *ASTIN Bulletin: The Journal of the IAA 49*(2), 433–455.

Liu, Q., C. Ling, and L. Peng (2019). Statistical inference for lee-carter mortality model and corresponding forecasts. *North American Actuarial Journal*, 1–29.
