# Supplementary material for: Future trends of life expectancy by education in the Netherlands
Source: BMC Public Health. 2022 Sep 2;22:1664. doi: 10.1186/s12889-022-13275-w (PMC9438160; doi:10.1186/s12889-022-13275-w)
Supplement: Supplementary file 3 — Additional file 3: Appendix 3. Life expectancy (LE) between age 35 and 80. [file 12889_2022_13275_MOESM3_ESM.pdf]

Supplementary material to "Future trends of life expectancy by education in the Netherlands"

by WJ Nusselder & AMB De Waegenaere et al, BMC Public Health 2022,

Appendix 3 Life expectancy (LE) between age 35 and 80

	Low	Change since 2018		Mid	Change since 2018		High	Change since 2018	
		Total	Per year		Total	Per year		Total	Per year
**Men**									
LE35-80									
2018	41.7			43.7			45.3		
2033	42.6	0.9	0.06	45.2	1.5	0.10	46.4	1.1	0.07
2048	43.4	1.7	0.06	46.3	2.6	0.09	47.2	1.9	0.06
**Women**									
LE35-80									
2018	44.1			45.8			46.6		
2033	44.3	0.4	0.02	46.7	0.9	0.06	47.3	0.7	0.04
2048	44.5	0.4	0.01	47.4	1.6	0.05	47.9	1.3	0.04
