# Supplementary material for: Future trends of life expectancy by education in the Netherlands
Source: BMC Public Health. 2022 Sep 2;22:1664. doi: 10.1186/s12889-022-13275-w (PMC9438160; doi:10.1186/s12889-022-13275-w)
Supplement: Supplementary file 4 — Additional file 4: Appendix 4. Life expectancies by calendar year. [file 12889_2022_13275_MOESM4_ESM.pdf]

Supplementary material to “Future trends of life expectancy by education in the Netherlands” by WJ Nusselder & AMB De Waegenaere et al, BMC Public Health 2022,

Appendix 4: Life expectancies by calendar year

Table A3-1 Life expectancy between age 35 and 85 and remaining life expectancy at age 35 and 65 by education and gender based on modelled mortality rates for period 2006 to 2048 for the Netherlands

	LE35-85						LE35						LE65					
	Men			Women			Men			Women			Men			Women		
	Low	Mid	High	Low	Mid	High	Low	Mid	High	Low	Mid	High	Low	Mid	High	Low	Mid	High
2006	40.4	42.1	44.3	43.8	45.1	46.0	41.8	43.9	47.2	46.8	48.9	50.1	16.0	17.1	19.3	19.8	21.2	21.8
2007	40.6	42.6	44.3	44.0	45.3	46.1	42.0	44.7	47.1	47.1	49.2	50.5	16.1	17.6	19.3	20.0	21.4	22.2
2008	41.1	42.3	44.3	43.8	45.5	46.0	42.7	44.1	47.1	46.8	49.4	50.4	16.5	17.2	19.3	19.9	21.7	22.2
2009	40.9	42.7	44.4	44.1	45.3	46.1	42.5	44.8	47.3	47.5	49.3	50.6	16.5	17.7	19.5	20.3	21.6	22.3
2010	41.0	42.9	44.5	44.1	45.6	45.5	42.6	45.0	47.5	47.5	49.7	48.5	16.5	17.8	19.6	20.3	21.9	20.4
2011	41.1	43.2	44.8	43.9	45.3	46.2	42.8	45.5	48.0	47.2	49.5	50.6	16.7	18.2	20.0	20.3	21.7	22.3
2012	41.3	43.1	44.8	43.9	45.4	46.3	43.1	45.4	47.8	47.1	49.6	51.0	16.9	18.1	19.9	20.2	21.8	22.7
2013	41.4	43.1	45.0	44.2	45.5	46.1	43.2	45.2	48.2	47.7	49.7	50.1	17.0	18.0	20.1	20.6	21.9	21.8
2014	41.6	43.5	45.1	44.2	45.4	46.4	43.5	45.9	48.3	47.8	49.8	51.1	17.2	18.5	20.3	20.8	21.9	22.7
2015	41.7	43.6	45.0	44.1	45.6	46.4	43.6	46.1	48.1	47.6	50.0	51.0	17.3	18.6	20.2	20.6	22.1	22.7
2016	41.4	43.8	45.1	44.0	45.5	46.4	43.1	46.4	48.4	47.3	49.8	50.6	17.1	18.8	20.3	20.5	21.9	22.3
2017	41.5	43.6	45.3	44.1	45.9	46.6	43.3	45.9	48.6	47.5	50.3	51.4	17.2	18.5	20.5	20.6	22.4	23.0
2018	41.7	43.7	45.3	44.1	45.8	46.6	43.6	46.0	48.7	47.5	50.3	51.5	17.3	18.5	20.5	20.7	22.3	23.0
2019	41.8	43.8	45.4	44.1	45.9	46.7	43.7	46.2	48.8	47.6	50.4	51.6	17.4	18.7	20.6	20.7	22.4	23.1
2020	41.8	44.1	45.5	44.2	46.0	46.7	43.7	46.6	48.9	47.7	50.6	51.4	17.5	19.0	20.7	20.9	22.6	22.9

2021	41.9	44.1	45.6	44.2	46.1	46.7	43.8	46.7	49.1	47.8	50.7	51.6	17.5	19.0	20.8	21.0	22.7	23.1
2022	41.9	44.2	45.7	44.2	46.1	46.8	43.9	46.8	49.2	47.9	50.9	51.7	17.6	19.1	20.9	21.0	22.8	23.2
2023	42.0	44.3	45.7	44.2	46.2	46.8	44.0	47.0	49.4	47.9	51.0	51.8	17.7	19.3	21.1	21.1	23.0	23.3
2024	42.0	44.4	45.8	44.2	46.2	46.9	44.1	47.1	49.5	48.0	51.2	52.0	17.8	19.4	21.2	21.2	23.1	23.4
2025	42.1	44.5	45.9	44.2	46.3	46.9	44.2	47.3	49.7	48.0	51.3	52.1	17.9	19.5	21.3	21.3	23.2	23.5
2026	42.2	44.6	45.9	44.3	46.4	47.0	44.3	47.4	49.8	48.1	51.5	52.2	18.0	19.6	21.4	21.4	23.3	23.7
2027	42.2	44.7	46.0	44.3	46.4	47.0	44.4	47.6	49.9	48.2	51.6	52.3	18.1	19.7	21.5	21.5	23.4	23.8
2028	42.3	44.8	46.1	44.3	46.5	47.1	44.5	47.7	50.1	48.2	51.7	52.5	18.2	19.8	21.6	21.5	23.6	23.9
2029	42.4	44.8	46.1	44.3	46.5	47.1	44.6	47.8	50.2	48.3	51.9	52.6	18.2	19.9	21.8	21.6	23.7	24.0
2030	42.4	44.9	46.2	44.3	46.6	47.2	44.5	48.0	50.4	48.1	52.0	52.7	18.1	20.0	21.9	21.4	23.8	24.1
2031	42.4	45.0	46.3	44.3	46.6	47.2	44.6	48.1	50.5	48.2	52.1	52.9	18.2	20.2	22.0	21.5	23.9	24.2
2032	42.5	45.1	46.3	44.3	46.7	47.3	44.7	48.3	50.6	48.3	52.3	53.0	18.3	20.3	22.1	21.6	24.0	24.3
2033	42.6	45.2	46.4	44.3	46.7	47.3	44.8	48.4	50.8	48.3	52.4	53.1	18.3	20.4	22.2	21.7	24.1	24.4
2034	42.6	45.2	46.5	44.3	46.8	47.3	44.9	48.6	50.9	48.4	52.6	53.2	18.4	20.5	22.3	21.8	24.3	24.6
2035	42.7	45.3	46.5	44.4	46.8	47.4	45.0	48.7	51.1	48.5	52.7	53.4	18.5	20.6	22.5	21.9	24.4	24.7
2036	42.7	45.4	46.6	44.4	46.9	47.4	45.1	48.8	51.2	48.5	52.8	53.5	18.6	20.7	22.6	21.9	24.5	24.8
2037	42.8	45.5	46.7	44.4	46.9	47.5	45.2	49.0	51.3	48.6	53.0	53.6	18.7	20.8	22.7	22.0	24.6	24.9
2038	42.9	45.6	46.7	44.4	47.0	47.5	45.3	49.1	51.5	48.7	53.1	53.8	18.8	20.9	22.8	22.1	24.7	25.0
2039	42.9	45.6	46.8	44.4	47.0	47.5	45.4	49.2	51.6	48.7	53.2	53.9	18.9	21.0	22.9	22.2	24.8	25.1
2040	43.0	45.7	46.8	44.4	47.1	47.6	45.5	49.4	51.7	48.8	53.4	54.0	19.0	21.1	23.0	22.3	25.0	25.2
2041	43.0	45.8	46.9	44.4	47.1	47.6	45.6	49.5	51.9	48.9	53.5	54.1	19.1	21.2	23.1	22.4	25.1	25.3
2042	43.1	45.8	46.9	44.4	47.2	47.7	45.7	49.6	52.0	48.9	53.6	54.3	19.1	21.3	23.3	22.5	25.2	25.5
2043	43.1	45.9	47.0	44.5	47.2	47.7	45.8	49.8	52.1	49.0	53.8	54.4	19.2	21.5	23.4	22.5	25.3	25.6
2044	43.2	46.0	47.0	44.5	47.3	47.7	45.9	49.9	52.3	49.1	53.9	54.5	19.3	21.6	23.5	22.6	25.4	25.7
2045	43.3	46.1	47.1	44.5	47.3	47.8	46.0	50.0	52.4	49.1	54.0	54.6	19.4	21.7	23.6	22.7	25.5	25.8
2046	43.3	46.1	47.1	44.5	47.3	47.8	46.1	50.2	52.5	49.2	54.2	54.8	19.5	21.8	23.7	22.8	25.7	25.9
2047	43.4	46.2	47.2	44.5	47.4	47.8	46.2	50.3	52.7	49.3	54.3	54.9	19.6	21.9	23.8	22.9	25.8	26.0
2048	43.4	46.3	47.2	44.5	47.4	47.9	46.3	50.4	52.8	49.3	54.4	55.0	19.7	22.0	23.9	23.0	25.9	26.1
