# Supplementary material for: Future trends of life expectancy by education in the Netherlands
Source: BMC Public Health. 2022 Sep 2;22:1664. doi: 10.1186/s12889-022-13275-w (PMC9438160; doi:10.1186/s12889-022-13275-w)

Supplementary material to "Future trends of life expectancy by education in the Netherlands" by

WJ Nusselder & AMB De Waegenaere et al, BMC Public Health 2022,

Appendix 5 Life expectancy between age 35 and 85 based on observed and modelled rates

Figure A4-1 Life expectancy between age 35 and 85 based on observed mortality rates (deaths/exposed) and modelled mortality rates, for men (upper panel) an for women (lower panel).

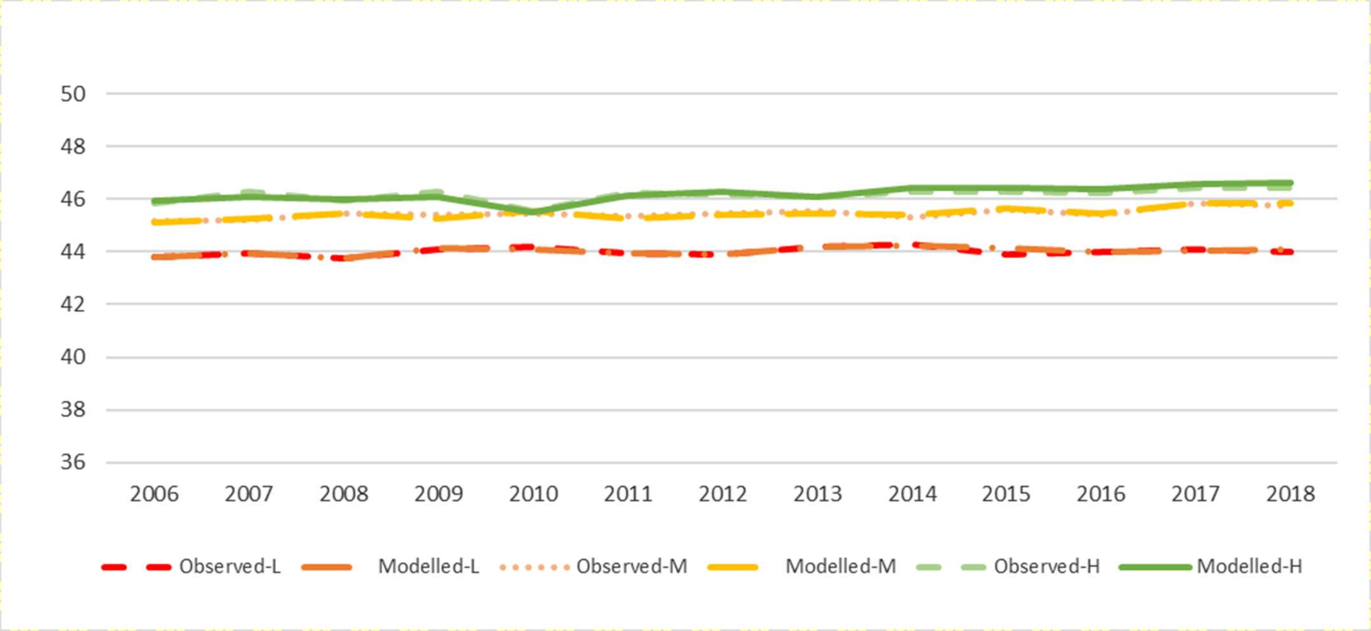

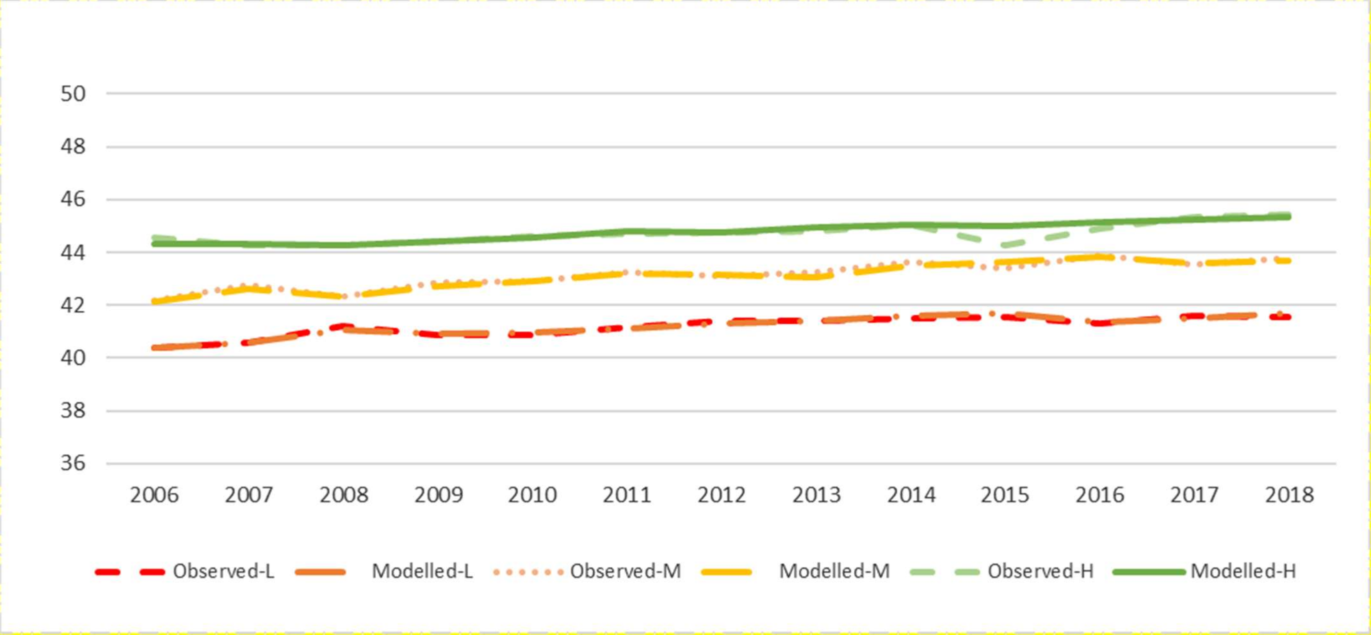

Supplement: Supplementary file 5 — Additional file 5: Appendix 5. Life expectancy between age 35 and 85 based on observed and modelled rates. [file 12889_2022_13275_MOESM5_ESM.pdf]
